# Prevalence and Correlates of Intestinal Parasites among Patients Admitted to Mirembe National Mental Health Hospital, Dodoma, Tanzania

**DOI:** 10.1155/2017/5651717

**Published:** 2017-05-22

**Authors:** Azan A. Nyundo, David Z. Munisi, Ainory P. Gesase

**Affiliations:** ^1^Department of Internal Medicine and Child Health, Psychiatry Division, School of Medicine, College of Health Science, The University of Dodoma, Dodoma, Tanzania; ^2^Department of Anatomy and Histology, College of Health Science, The University of Dodoma, Dodoma, Tanzania; ^3^Department of Microbiology and Parasitology, College of Health Sciences, The University of Dodoma, Dodoma, Tanzania

## Abstract

**Background:**

Neglected tropical diseases continue to be one of the leading causes of morbidity and mortality in the developing world. Psychiatric patients are among groups at risk for parasitic infection although control and monitoring programs largely overlook this population. This study aimed at determining prevalence and factors associated with intestinal parasitic infection among patients admitted to a psychiatric facility.

**Method:**

The study followed cross-sectional design; all the residing patients that met the inclusion criteria were included in the survey. Stool samples were collected and examined by direct wet preparation and formol-ether concentration. Data were analyzed with STATA version 12.1; Chi-square test was computed to determine the level of significance at *p* value < 0.05.

**Results:**

Of all 233 patients who returned the stool samples, 29 (12.45%) screened were positive for an intestinal parasite. There was no significant association between parasite carriage and age, sex, or duration of hospital stay.

**Conclusion:**

The study shows that intestinal parasitic infection is common among patients in a psychiatric facility and highlights that parasitic infections that enter through skin penetration may be a more common mode of transmission than the oral route. Furthermore, the study underscores the need for surveillance and intervention programs to control and manage these infections.

## 1. Introduction

Parasitic infections remain a significant cause of morbidity and mortality in the developing world [1–4]. Socioeconomic and environmental factors such as poor personal hygiene, lack of access to clean water, sanitation, and overcrowding have all been associated with intestinal parasitic infections [3]. The infected individual may have overall impairment in survival, growth, nutritional status, cognitive performance, and a scholastic achievement [5].

Although the high prevalence of parasitic infection has been reported in psychiatric facilities worldwide, little is known about the status in Tanzanian settings. The most recent WHO report shows that, in Tanzania, neuropsychiatric disorders are estimated to contribute to 5.3% of global burden of disease [6]. Despite the high burden of mental illness in the country, there are only 1700 beds reserved for psychiatric patients, 700 in the only psychiatric hospital in the country, and 662 in the general hospitals and community health facilities. Furthermore, the burden is overwhelming for the mental health profession whereby, for a population of 100000, there are 0.04 psychiatrists, 0.007 psychologists, 0.01 social workers, 0.009 occupational therapists, and an unknown number of psychiatric nurses [6].

By the nature of their illnesses and consequently poor hygiene, psychiatric patients are at a relatively higher risk for parasitic infection [7, 8]. Psychiatric disorders contribute to 5.3% of global burden of disease, and, for the case of Tanzania, there is a huge gap between resources such as workforce, facilities, and patients needing the services [6] which may lead to overcrowding and poor patient care. The characteristics of patients in the mental hospital such as mobility, poorly cooperating with hygienic measures, and readily interacting with each other make transmission of infections easy [9]. In these settings, the prevalence rate of intestinal parasitic infections has been reported to range from 5% to 35.2% or even more [10–12]. The Society for Healthcare Epidemiology of America/Association for Professionals in Infection Control and Epidemiology has provided guidelines on infection prevention and control in long-term care facilities; however, the characteristics of psychiatric patients make it difficult to implement these recommendations [9, 13, 14]. Intestinal parasitic infections have been associated with undernutrition and anemia and the severity of which may depend on coexisting disease conditions [15, 16]. It is further known that undernutrition may increase susceptibility to other infection. Therefore, treatment of parasitic infections may spell a better prognosis for psychiatric patients [17].

For the implementation of any proposed intervention, it is important to determine the magnitude of intestinal parasitic infections in the psychiatric hospital. This study aims to investigate the carriage of intestinal parasitic infections among residents of Mirembe Hospital which is the only tertiary psychiatric hospital in the country.

## 2. Methods

### 2.1. Study Design and Settings

The study was a cross-sectional design conducted at Mirembe National Psychiatric Referral Hospital located in Dodoma region, the capital city of Tanzania.

The hospital has a total population ranging between 300 and 400 in-patients at one point in time. The hospital admits adult male and female patients suffering from various psychiatric conditions including schizophrenia, bipolar disorders, substance use disorders, and epileptic psychosis patients. The majority of the patients are males and constitute at least 60 percent of all patients in the ward.

### 2.2. Study Population

The study included patients that were admitted to Mirembe during the time of study and met the inclusion criteria. Informed consent was sought from caregivers for participants who could not provide consent. Patients who were within two weeks of active treatment with antiparasitic drugs were excluded from the study. All participants that were in the ward during the study period were enrolled and then tested for parasitic infection. The analysis excluded all the patients who did not return the stool samples.

### 2.3. Data Collection and Management

A researcher-designed questionnaire specifically captured sociodemographic and clinical characteristics of the patients. Data collection focused on the variables of interest that were retrieved from the patient's case files; this included age, sex, and duration of stay in the hospital. From each participant, we collected a single freshly voided stool sample in a well labeled wide mouth stool container. The stool was then immediately processed using direct technique (saline and iodine mounts) to identify trophozoite and cysts of protozoan parasites and using formol-ether concentration technique to detect eggs and larva of intestinal helminths and* Schistosoma mansoni*.

### 2.4. Data Analysis

The data was entered into Microsoft Excel and analyzed with STATA version 12.1 (StataCorp, Texas). Variables of interest were summarized into frequencies, proportions, mean, and confidence intervals. Pearson Chi-square test and Fisher's exact test were computed to determine the association between variables; the level of significance was set at *α* ≤ 0.05.

### 2.5. Ethical Consideration

Permission to conduct the research at the hospital premises was sought from hospital management. Informed consent was secured from caretakers or relatives of patients in no mental or physical capacity to do so. Patients/caretakers were fully informed that participation is voluntary and they may opt to stop participating at any point of the study without having to face any consequences.

## 3. Results

### 3.1. Demographic Characteristics of the Study Participants

In total, 363 patients were involved in this study. The age range of the study participants was 12–69 years with a mean of 32.7 ± 10.9 years of whom the majority were males (273) (75.21%). Almost equal number of participants (170 (46.83%) and 167 (46.01%)) were within the 12–29 and 30–49 years of age ranges, respectively; the remaining were of the age between 50 and 69 years (Table 1).

### 3.2. Distribution of Psychiatric Diagnosis among Participants

Of all 363 participants, the majority (230) (63.4%) suffered from schizophrenia spectrum disorders followed by substance-related disorders with 58 (16%) of the admitted patients. Other conditions included epilepsy and related disorders (9.1%), bipolar mood disorder (4.7%), acute brief psychosis (3.9%), and organic psychosis or related disorders (3.0%). With exception to acute brief psychosis and organic psychosis of which gender was evenly distributed, the majority of other diagnosis were overwhelmed by male predominance (Table 2).

### 3.3. Prevalence of Intestinal Parasitic Infections among Study Participants

Of all the study participants, 233 provided a stool sample for parasitological examination. Overall, 29 (12.45%, 95% CI: 8.21%–16.69%) of the study participants were infected with at least one of the investigated intestinal parasitic infections. The most common intestinal parasites found were* Schistosoma mansoni* and hookworm which constituted 41.38% and 37.93% of all the participants with positive results (29) (Table 3).

### 3.4. Relationship between Intestinal Parasitic Infections and Demographic Characteristic of the Study Participants

Intestinal parasitic infections were computed against age, sex, and duration of hospital stay. It was observed that male patients were more infected than female patients, though the observed difference was not statistically significant (*p* > 0.05). Likewise, infections were more common in younger patients than older patients, but again the observed difference was not statistically significant (*p* > 0.05) (Table 4).

## 4. Discussion

Previous reports elsewhere have indicated that intestinal parasitic infections inflict a significant morbidity among individuals in mental institutions [18, 19]. The present study investigated the prevalence and factors associated with intestinal parasitic infections among patients admitted to a tertiary psychiatric facility in Tanzania.

As for the patient's profile, the study revealed that the majority (79%) of participants were males suffering from either schizophrenia or substance-related disorders; this may support the notion that males use psychiatric services more often than females [20]. The fact that the admitted male patients were relatively younger compared to female counterparts is in line with the well documented natural course of schizophrenia of having an onset at a relatively younger age among males than females counterparts [21, 22] usually accompanied by poor prognosis among the males with regular admissions and longer duration of hospital stay [23]. Concurrently, males with mental disorders are usually more afflicted with substance-related disorders than females [24] which is also a factor that is associated with the poor social function, medication noncompliance, symptom exacerbation, frequent hospitalization, and poor treatment response [25, 26]. Although not statistically significant, males were potentially more likely to be infected than females; however, the uneven distribution between the two genders makes their comparison statistically less valid. Again though not statistically significant, intestinal parasitic infections appeared to be more common among young patients and they decreased towards older patients. This observation is in line with a common knowledge on the epidemiology of intestinal parasitic infections having peak prevalence among adolescents and young adults [2].

Little is known about the prevalence of intestinal parasitic infection among the adult population in Tanzania as most studies have been conducted on school-age population; however, the nature of psychiatric diagnosis and the overall settings of psychiatric facilities and illnesses poses the risks for intestinal parasitic infection for this population [9]. In this study, the majority of species identified were all related in the mode of transmission.* Schistosoma mansoni*, hookworm, and* Strongyloides stercoralis* which constitute about 83% of the identified infections enter through skin penetration whereas* Trichuris trichiura* and* Entamoeba histolytica*/*dispar* enter through the mouth.

The diagnosis of* S. mansoni* with no single patient screened positive for* S. haematobium* in this study supports the earlier observations that* S. mansoni* is more distributed in the central part of Tanzania including Dodoma [27–31] where the study was conducted; however, schistosomiasis is considered to be endemic throughout the country. The most recent report of 2012 indicates that 51.5% of the approximately 44 million Tanzanians are infected with schistosomiasis [32]; this suggests that the whole population is at risk of schistosoma infection. However the predominance of* S. mansoni* among the detected infections may also be a result of treatment of all patients upon admission, irrespective of their infection status, with albendazole as reported by the medical officer in charge (verbal communication). As albendazole is not effective against* S. mansoni* and there is no water body within the hospital premises that could harbour snail intermediate host for the parasite to have been acquired after admission, it is likely that the infections with this parasite were acquired before admission. This highlights the need to screen for intestinal parasites for all admitted patients so that specific treatment is offered upon admission.

Again, since albendazole which was offered at admission is efficacious to hookworms, the observed high prevalence hookworm infection in our study may signify infections acquired after admission. This correlates with documented risk factors which are common among patients admitted to psychiatric hospitals such as poor personal hygiene [33–36], poor sanitation [34, 37], and exposure to soil with filariform larvae that penetrate the skin [35, 38, 39]. Psychiatric patients in the study site tend to walk around bare-footed, the majority of them with poor personal care including poor defecation practices which are the common risks for hookworm infection [25–29].* Strongyloides stercoralis* was another parasite that enters through skin penetration; however, there was only one person who was infected with* Strongyloides stercoralis*, and, moreover, the infected individual was infected just five days after admission and the offered albendazole is less effective with* Strongyloides stercoralis* [40]; this highly suggests that the patient brought the infection into the facility further stressing the need to screen for intestinal parasitosis and offering appropriate treatment upon admission.

The WHO ranks* Entamoeba histolytica*/*dispar* and* Trichuris trichiura* among prioritized food-borne intestinal parasites with the substantive burden of disease [41]. A relatively low prevalence of 3/263 (1.1%) and 1/263 (0.4%) for* Entamoeba histolytica*/*dispar* and* Trichuris trichiura*, respectively, may suggest that these parasites may not be as prevalent in the study area despite the patients being at risk. Conversely, the institution has a standard operating procedure to ensure early detection and prompt management once the patient shows symptoms and signs of diarrheal diseases and in some occasions the sufferers may be secluded to diarrheal ward if necessary. The institution also frequently deworms them with albendazole; this may explain why no single patient screened was positive for* Ascaris lumbricoides* and showed very low prevalence of* Trichuris trichiura*.

## 5. Study Limitations

The cross-sectional nature of the study makes the causal relationship between the dependent and independent factors limited. Furthermore, the lack of control group limits the generalizability of the study findings within patients in the psychiatric population.

## 6. Conclusion

This study highlights the presence of parasitic infections, offers room for related complications, and underscores that the nature of the psychiatric patients and facilities, in general, is potentially at risk for rapid spread should the outbreak occur. The study eludes the effectiveness of surveillance programs and encourages the practice of screening and deworming programs upon admission to combat the spread and manage specific parasitic infections among patients in psychiatric hospitals.

## Figures and Tables

**Table 1:** Demographic characteristics of the study respondents.

Age category	Sex		Total *n* (%)
	Female *n* (%)	Male *n* (%)	
12–29	26 (28.89)	144 (52.75)	170 (46.83)
30–49	51 (56.67)	116 (42.49)	167 (46.01)
50–69	13 (14.44)	13 (4.76)	26 (7.16)

*Total*	90 (100.00)	273 (100.00)	363 (100.00)

**Table 2:** Psychiatric diagnosis by gender distribution.

Working diagnosis	Sex		Frequency (%/363)
	Female (%)	Male (%)	
Acute brief psychosis	7 (50.00)	7 (50.00)	14 (3.86)
Substance-related disorders	5 (8.62)	53 (91.38)	58 (15.98)
Bipolar mood disorders	4 (23.53)	13 (76.47)	17 (4.68)
Epilepsy-related disorders	7 (21.21)	26 (78.76)	33 (9.09)
Schizophrenia spectrum disorders	61 (26.32)	169 (73.48)	230 (63.36)
Organic psychosis and related disorders	6 (54.55)	5 (45.45)	11 (3.03)

*Total*	*90 (24.79)*	*273 (75.21)*	*363 (100)*

**Table 3:** Intestinal parasites found following stool examination.

Parasite	Frequency	Percent (%)	95% CI
*Schistosoma mansoni*	12	41.38	23.45%–59.31%
Hookworm	11	37.93	20.27%–55.59%
*Entamoeba histolytica*/*dispar*	3	10.34	**0.74%–21.42%**
*Trichuris trichiura*	2	6.90	**2.32%–16.12%**
*Strongyloides stercoralis*	1	3.45	**3.19%–10.09%**

*Total*	29	100	

**Table 4:** Association between intestinal parasitic infections and participant's sex, age, and duration of hospitalization (in days).

Characteristic	Infection status		Total *n* (%)	*p value*
	Negative *n* (%)	Positive *n* (%)		
*Sex*				
Female	49 (24.02)	5 (17.24)	54 (23.18)	0.418^*∗*^
Male	155 (75.98)	24 (82.76)	179 (76.82)	
*Age *				
12–29	93 (45.59)	20 (68.97)	113 (48.50)	0.070^*∗∗*^
30–49	96 (47.06)	8 (27.59)	104 (44.64)	
50–69	15 (7.35)	1 (3.45)	16 (6.87)	
*Hospitalization days*				
1–30	102 (50.00)	14 (48.28)	116 (49.79)	0.984^*∗*^
31–60	47 (23.04)	7 (24.14)	54 (23.18)	
>60	55 (26.96)	8 (27.59)	63 (27.04)	

*Total *	*204 (100.00)*	*29 (100.00)*	*233 (100.00)*	

^*∗*^*p* value based on chi-square statistic and  ^*∗∗*^Fisher exact test.

## References

[1] WHO World Health Assembly documents. http://www.who.int/schistosomiasis/resources/wha/en/.

[2] Hotez P. J., Bundy D. A. P., Beegle K., Jamison D. T., Breman J. G., Measham AR. (2006). Helminth infections: soil-transmitted helminth infections and schistosomiasis. *Disease Control Priorities in Developing Countries*.

[3] WHO Expert Committee (2002). *Prevention and Control of Schistosomiasis and Soil-Transmitted Helminthiasis*.

[4] Noyer C. M., Brandt L. J. (1999). Parasitic infections of the gastrointestinal tract. *Current Gastroenterology Reports*.

[5] de Silva N. R., Guyatt H. L., Bundy D. A. P. (1997). Morbidity and mortality due to Ascaris-induced intestinal obstruction. *Transactions of the Royal Society of Tropical Medicine and Hygiene*.

[6] WHO United Republic of Tanzania: country profiles. http://www.who.int/gho/countries/tza/country_profiles/en/.

[7] Giacometti A., Cirioni O., Balducci M. (1997). Epidemiologic features of intestinal parasitic infections in Italian mental institutions. *European Journal of Epidemiology*.

[8] Sirivichayakul C., Pojjaroen-Anant C., Wisetsing P., Siripanth C., Chanthavanich P., Pengsaa K. (June 2003). *Prevalence of intestinal parasitic infection among Thai people with mental handicaps*.

[9] Fukuta Y., Muder R. R. (2013). Infections in psychiatric facilities, with an emphasis on outbreaks. *Infection Control and Hospital Epidemiology*.

[10] Duedu K. O., Karikari Y. A., Attah S. K., Ayeh-Kumi P. F. (2015). Prevalence of intestinal parasites among patients of a Ghanaian psychiatry hospital. *BMC Research Notes*.

[11] Tappeh K. H., Mohammadzadeh H., Rahim R. N., Barazesh A., Khashaveh S., Taherkhani H. (2010). Prevalence of intestinal parasitic infections among mentally disabled children and adults of Urmia, Iran. *Iranian Journal of Parasitology*.

[12] Gatti S., Lopes R., Cevini C. (2000). Intestinal parasitic infections in an institution for the mentally retarded. *Annals of Tropical Medicine & Parasitology*.

[13] Smith P. W., Bennett G., Bradley S. (2008). SHEA/APIC Guideline: infection prevention and control in the long-term care facility. *American Journal of Infection Control*.

[14] Smith P. W., Bennett G., Bradley S. (2008). SHEA/APIC guideline: infection prevention and control in the long-term care facility. *Infection Control and Hospital Epidemiology*.

[15] Brito L. L., Barreto M. L., Silva R. D. C. R. (2006). Moderate- and low-intensity co-infections by intestinal helminths and schistosoma mansoni, dietary iron intake, and anemia in brazilian children. *American Journal of Tropical Medicine and Hygiene*.

[16] Nyarango R. M., Aloo P. A., Kabiru E. W., Nyanchongi B. O. (2008). The risk of pathogenic intestinal parasite infections in Kisii Municipality, Kenya. *BMC Public Health*.

[17] Katona P., Katona-Apte J. (2008). The interaction between nutrition and infection. *Clinical Infectious Diseases*.

[18] Huminer D., Symon K., Groskopf I. (Mar 1992). Seroepidemiologic study of toxocariasis and strongyloidiasis in institutionalized mentally retarded adults. *American Journal of Tropical Medicine and Hygiene*.

[19] Braun T. I., Fekete T., Lynch A. (1988). Strongyloidiasis in an institution for mentally retarded adults. *Archives of Internal Medicine*.

[20] Shapiro S., Skinner E. A., Kessler L. G. (1984). Utilization of Health and Mental Health Services: Three Epidemiologic Catchment Area Sites. *Archives of General Psychiatry*.

[21] Angermeyer M. C., Kühn L. (1988). Gender differences in age at onset of schizophrenia: an overview. *European Archives of Psychiatry and Neurological Sciences*.

[22] Kirkbride J. B., Fearon P., Morgan C. (2006). Heterogeneity in incidence rates of schizophrenia and other psychotic syndromes: findings from the 3-center ÆSOP study. *Archives of General Psychiatry*.

[23] Kao Y.-C., Liu Y.-P. (2010). Effects of age of onset on clinical characteristics in schizophrenia spectrum disorders. *BMC Psychiatry*.

[24] Brady K. T., Sinha R. (2005). Co-occurring mental and substance use disorders: the neurobiological effects of chronic stress. *American Journal of Psychiatry*.

[25] Brady K., Anton R., Ballenger J. C., Bruce Lydiard R., Adinoff B., Selander J. (1990). Cocaine abuse among schizophrenic patients. *American Journal of Psychiatry*.

[26] Dixon L., Haas G., Weiden P., Sweeney J., Frances A. (1990). Acute effects of drug abuse in schizophrenic patients: clinical observations and patients' self-reports. *Schizophrenia Bulletin*.

[27] Webbe G. (1959). A bilharzia and molluscan survey in the Handeni and Korogwe districts of Tanganyika. *Journal of Tropical Medicine and Hygiene*.

[28] Magendantz M. (1972). The biology of biomphalaria choanomphala and b. sudanica in relation to their role in the transmission of Schistosoma mansoni in Lake Victoria at Mwanza, Tanzania.. *Bulletin of the World Health Organization*.

[29] Wydell S. H. (1958). Some abdominal complications of S. mansoni as seen on Ukerewe Island. *East African Medical Journal*.

[30] Brooker S., Kabatereine N. B., Smith J. L. (2009). An updated atlas of human helminth infections: the example of East Africa. *International Journal of Health Geographics*.

[31] Jordan P., Webbe G., Sturrock R. F. (1993). *Human schistosomiasis*.

[32] Rollinson D., Knopp S., Levitz S. (2013). Time to set the agenda for schistosomiasis elimination. *Acta Tropica*.

[33] Gunawardena G. S. A., Karunaweera N. D., Ismail M. M. (2005). Effects of climatic, socio-economic and behavioural factors on the transmission of hookworm (Necator americanus) on two low-country plantations in Sri Lanka. *Annals of Tropical Medicine and Parasitology*.

[34] Olsen A., Samuelsen H., Onyango-Ouma W. (2001). A study of risk factors for intestinal helminth infections using epidemiological and anthropological approaches. *Journal of Biosocial Science*.

[35] Traub R. J., Robertson I. D., Irwin P., Mencke N., Thompson R. C. A. (2004). The prevalence, intensities and risk factors associated with geohelminth infection in tea-growing communities of Assam, India. *Tropical Medicine and International Health*.

[36] Asaolu S. O., Ofoezie I. E. (2003). The role of health education and sanitation in the control of helminth infections. *Acta Tropica*.

[37] Ensink J. H. J., van der Hoek W., Mukhtar M., Tahir Z., Amerasinghe F. P. (2005). High risk of hookworm infection among wastewater farmers in Pakistan. *Transactions of the Royal Society of Tropical Medicine and Hygiene*.

[38] Liabsuetrakul T., Chaikongkeit P., Korviwattanagarn S. (2009). Epidemiology and the effect of treatment of soil-transmitted helminthiasis in pregnant women in Southern Thailand. *Southeast Asian Journal of Tropical Medicine and Public Health*.

[39] Tomono N., Anantaphruti M. T., Jongsuksuntigul P. Risk factors of helminthiases among schoolchildren in southern Thailand. http://imsear.hellis.org/handle/123456789/35891.

[40] Henriquez-Camacho C., Gotuzzo E., Echevarria J. (2016). Ivermectin versus albendazole or thiabendazole for Strongyloides stercoralis infection. *Cochrane Database of Systematic Reviews*.

[41] World Health Organization; Department of Food Safety; Zoonoses and Foodborne Diseases First formal meeting of the Foodborne Disease Burden Epidemiology Reference Group (FERG) : implementing strategy, setting priorities and assigning the tasks. http://www.who.int/iris/handle/10665/43905.

